# The Proportion of ALDEFLUOR-Positive Cancer Stem Cells Changes with Cell Culture Density Due to the Expression of Different ALDH Isoforms

**DOI:** 10.17140/CSMMOJ-2-113

**Published:** 2015-12-09

**Authors:** Lynn M. Opdenaker, Shirin R. Modarai, Bruce M. Boman

**Affiliations:** 1 Department of Biological Sciences, University of Delaware, 118 Wolf Hall, Newark, DE 19716, USA; 2 Center for Translational Cancer Research, Helen F. Graham Cancer Center and Research Institute, 4701 Ogletown-Stanton Rd, Newark, DE 19713, USA

**Keywords:** ALDEFLUOR, Cancer stem cells, ALDH isoforms, Density

## Abstract

A significant number of discrepancies exist within the literature regarding ALDEFLUOR-positive stem cell populations in cell lines. We hypothesized that these inconsistencies resulted from differences in culture conditions, particularly cell density. We cultured several colon cancer cell lines (N=8) at high and low densities and found a significant decrease in ALDEFLUOR-positive cell populations at high density. However, we found no changes in the CD166-positive stem cell population, self-renewal, or cell cycle distribution of cells cultured at different densities. Interestingly, when we sorted both ALDEFLUOR positive and negative populations from the different density cultures, we identified a significant number of Aldehyde dehydrogenase (ALDH) isoforms whose expression was decreased in ALDEFLUOR-positive stem cells cultured at high density. This novel finding suggests that multiple ALDH isoforms contribute to ALDEFLUOR activity in colon cancer stem cells and decreases in ALDEFLUOR-positive stem cells at high cell density are due to decreased expression of multiple ALDH isoforms. Thus, designing therapeutics to target ALDEFLUOR-positive cancer stem cells may require inhibition of multiple ALDH isoforms.

## INTRODUCTION

With the increased use of the ALDEFLUOR assay to isolate and identify Cancer Stem Cells (CSCs), we noticed different published papers reported different percent ALDEFLUOR-positive cells for the same cancer cell lines.^1–4^ This difference was also seen in our own cell cultures especially when the colon cancer cell lines were grown in low or high densities. We hypothesized that the variation in ALDEFLUOR results was due to cultures being grown at different densities and that ALDEFLUOR-positive stem cell populations might express different Aldehyde dehydrogenase (ALDH) isoforms dependent on culture density. There is a paucity of data reported on cell culture density in papers giving results on the number of CSCs using the ALDEFLUOR assay. However, there are a few articles and reviews that acknowledge that gene expression can change with respect to the changing characteristics of stem cells observed at different culture densities.^5–9^

Aldehyde dehydrogenase (ALDH) is part of a family of enzymes localized in the cytoplasm, mitochondria or nucleus.^10^ In recent years, ALDH has been used to identify CSCs in various solid tumors and it has become a universal marker for CSCs in epithelial cancers.^10,11^ The ALDEFLUOR assay allows for the isolation of viable CSCs from patient tissue samples and for further analysis involving *in vitro* and *in vivo* studies. This assay measures the ALDH enzyme activity *via* cleavage of a fluorescent substrate, BODIPY-Aminoacetaldehyde (BAAA), that consists of an aminoacetaldehyde moiety bonded to the BODIPY fluorochrome.^12^ To measure the exact percent of cells with high ALDH activity, an ALDH inhibitor, Diethylaminobenzaldehyde (DEAB), is used as a control.

There are 19 known isoforms of ALDH and several have been implicated in different types of cancers. Isoform expression is cell and tissue type dependent. In breast cancer, ALDH1, ALDH1A1, 1A3, and 3A1 have all been identified and correlated with aggression, progression, or poor survival.^13–16^ ALDH1A3 has also been identified as the main isoform responsible for ALDEFLUOR activity in breast cancer cells.^17^ In ovarian cancer, ALDH1A1 overexpression was tumor-type specific. Overexpression of isoforms 1A3, 3A2 and 7A1 in ovarian cancer appear to be a more consistent finding.^18^ ALDH7A1 overexpression is reported to contribute to metastasis in prostate cancer.^19^ Overexpression of ALDH3A1 has also been identified in prostate cancer and hepatocellular carcinoma. In colon, ALDH1B1 was identified as a potential CSC biomarker in patient samples.^20^ Particularly in colon, a pan-ALDH1 antibody has been used to identify expression patterns. Expression of the ALDH1 family in colon cancer cell lines and patient samples have been utilized by several lab groups to identify and isolate CSCs.^2,11,21,22^ While many isoforms have been identified as biomarkers and indicators of tumorigenicity and cancer progression, several other isoforms still remain to be investigated.

Each of the different ALDH isoforms has a particular specificity for different substrates linked to their role in cellular function. For example, ALDH1A1, ALDH1A2, ALDH1A3 and ALDH8A1 have been linked to retinoic acid cell signaling *via* retinoic acid production due to the oxidation of All-Trans-Retinoic Acid (ATRA) and 9-cis retinoic acid.^23^ The other isoforms are not directly related to retinoic acid signaling and instead have slightly different roles. Their substrate preferences also depend on the intra-cellular location of the isoforms, as some isoforms are mitochondrial and others are found in the cytoplasm.

In our current study, we analyzed colon cancer cell lines cultured in low and high densities to ascertain the effects of density on the ALDH population size *via* the ALDEFLUOR assay. Our goal was to investigate how density culture conditions contribute to changes to the ALDEFLUOR cell population size and whether or not this regulation occurs due to changes in a specific ALDH isoform, particularly one that might be unique to colon cancer cells. To our knowledge, this is the first report of density differences observed with the ALDEFLUOR assay and an attempt of looking at the possible causes behind these differences.

## METHODS

### Cell Culture

HT29 and HCT116 cells obtained from American Type Culture Collection (ATCC; Manassas, VA, USA) were grown in monolayer cultures and maintained in: McCoys medium (GIBCO/Life Technologies) supplemented with 5% Fetal Bovine Serum (FBS) and 100 units/ml penicillin and 100 ug/ml streptomycin (P/S). SW480 cells obtained from ATCC were maintained in Leibovitz’s 15 (L-15) medium (GIBCO/Life Technologies) supplemented with 5% FBS and P/S. LoVo, Colo320 and DiFi cells were maintained in Roswell Park Memorial Institute (RPMI-1640) medium (GIBCO/Life Technologies) supplemented with 5% FBS and P/S. Hepatocellular carcinoma (HepG2) and Human embryonic kidney cells (HEK293) cell lines were maintained in Dulbecco’s Modified Eagle’s medium (DMEM) supplemented with 5% FBS and P/S. All cell cultures were maintained at 37 °C in humidified air at 5% CO_2_. To achieve the desired low and high cell densities, cells were plated at 400,000 cells/100 mm culture dish (Greiner, VWR International) for low density and 800,000 cells/100 mm dishes for high density. Cells were allowed to grow for 3–5 days until a confluency of 30–40% was achieved for low density and 70–80% was achieved for high density. Culture medium for all cell lines was changed every 48 hours. Cell cultures never reached full confluency at the time of analysis. All experiments in this study were conducted within ten passages.

### ALDEFLUOR Assay

Protocol was followed according to the manufacturer (STEMCELL Technologies). Briefly, cells were grown to 80% confluence and lifted using 0.25% Trypsin-EDTA (Fisher Scientific). Cells were spun for five minutes to pellet and washed once with PBS. Cells were resuspended in ALDEFLUOR assay buffer at a concentration of one million cells/ml. Two tubes were labeled as control and sample. To the control tube, 5 µl of the DEAB inhibitor was added. To the sample tube, 5 µl of the activated ALDEFLUOR reagent was added, mixed and immediately 500 µl of the suspension was taken out and put in the control tube with the inhibitor. Cells were incubated for 40 minutes at 37 °C. After incubation, cells were spun for five minutes to pellet and washed once with ALDEFLUOR buffer. Cell were resuspended in 500 µl ALDEFLUOR buffer and passed through a BD round bottom tube with a 50 µm cell strainer (BD Biosciences). Samples were placed on ice and covered from light until ready for analysis on the BD FACSAria II Flow Cytometer.

### Flow Cytometry

All cells were grown to the appropriate low and high culture densities and lifted using an (Ethylenedinitrilo)tetraacetic acid (EDTA) based solution called Cell Stripper (Fisher Scientific). Cells were spun for five minutes to pellet and resuspended in a 3% BSA blocking solution made in PBS. Cells were incubated for 1 hour on ice in this blocking solution before 5 µl of CD166-PE conjugated antibody (BD Biosciences) was added to the cells. An appropriate PE conjugated IgG control (BD Biosciences) was used at an equal concentration to the CD166 antibody. Cells were incubated on ice for 30 minutes. Following primary antibody and IgG incubation, cells were washed twice with PBS, and then resuspended in PBS. Cell suspensions were passed through a BD round bottom tube with a 50 µm cell strainer (BD Biosciences). Cell surface staining was analyzed using the BD FACSAria II Flow Cytometer.

### Colonosphere Assay

Cells were plated at a cell density of 200 cells per 100 µl of stem cell media which is composed of serum free DMEM/F12 (GIBCO Inc.) with the addition of Epidermal Growth Factor (EGF) and basic Fibroblast Growth Factor (bFGF) and B-27 complex without Vitamin A (Life Technologies, Carlsbad, CA, USA). The method and culture medium used to perform the colonosphere assay was from a previously published article.^24^ Ultra low attachment plates (BD Biosciences) were used for this assay and colon spheres were analyzed for their size (diameter) and numbers per well on day ten using the 10× objective of a phase contrast microscope.

### Cell Cycle

Cells were plated at low and high densities in 100 mm cell culture dishes and harvested using trypsin. The cells were washed two times with PBS and then fixed in 1 mL ice cold 70% ethanol. Ethanol was added dropwise to the cells while vortexing to avoid clumping. Cells were fixed for at least 48 hours before analysis and stored in 4 °C until ready to stain with propidium iodide. When ready to stain and analyze samples, fixed cells were washed twice with PBS and spun down at 2000 rpm for 5 minutes each time. Cells were resuspended in 1mL Fx-Cycle PI/RNase staining solution (Invitrogen) and allowed to incubate for 15 minutes at room temperature and covered from light. Cells were transferred to a BD tube and analyzed on the BD FACSAria II Flow Cytometer with the PE channel.

### Reverse Transcriptase-Polymerase Chain Reaction

Ribonucleic acid (RNA) was isolated from ALDEFLUOR positive and negative sorted cells that were cultured at high and low densities. RNA was isolated using the TRIzol reagent (Thermo Fisher Scientific) and the protocol provided by the manufacturer. Briefly, cells were pelleted and lysed in TRIzol reagent. After a brief incubation at RT, chloroform was added and samples were incubated for 2–3 minutes at RT. Samples were centrifuged and the aqueous phase was removed. RNA was precipitated with isopropyl alcohol, washed with ethanol and resuspended in sterile water. RNA was Deoxyribonuclease (DNAse) treated with the Deoxyribonucleic acid (DNA)-free DNA Removal Kit (Ambion) per the manufacturer’s protocol. Concentration of the RNA was determined using the Tecan Group Ltd. (TECAN) Infinite 200 PRO microplate reader. Equal amounts of RNA were used for the reverse transcriptase reaction. Using the SuperScript III First-Strand Synthesis System (Life Technologies) and the provided protocol, complementary DNA (Cdna) was generated. Polymerase Chain Reaction (PCR) was performed using 50 ng cDNA and the GoTaq Green PCR Mastermix (Promega). Primers and reaction conditions for the 19 ALDH isoforms were previously published.^17^ PCR products were analyzed on a 1.5% agarose gel and imaged on the Syngene imaging system.

### Statistics

All statistics were performed using Student’s *t*-test using Microsoft excel or a Paired *t*-test using Graph Pad Prism software analysis.

## RESULTS

**Figure 1:**
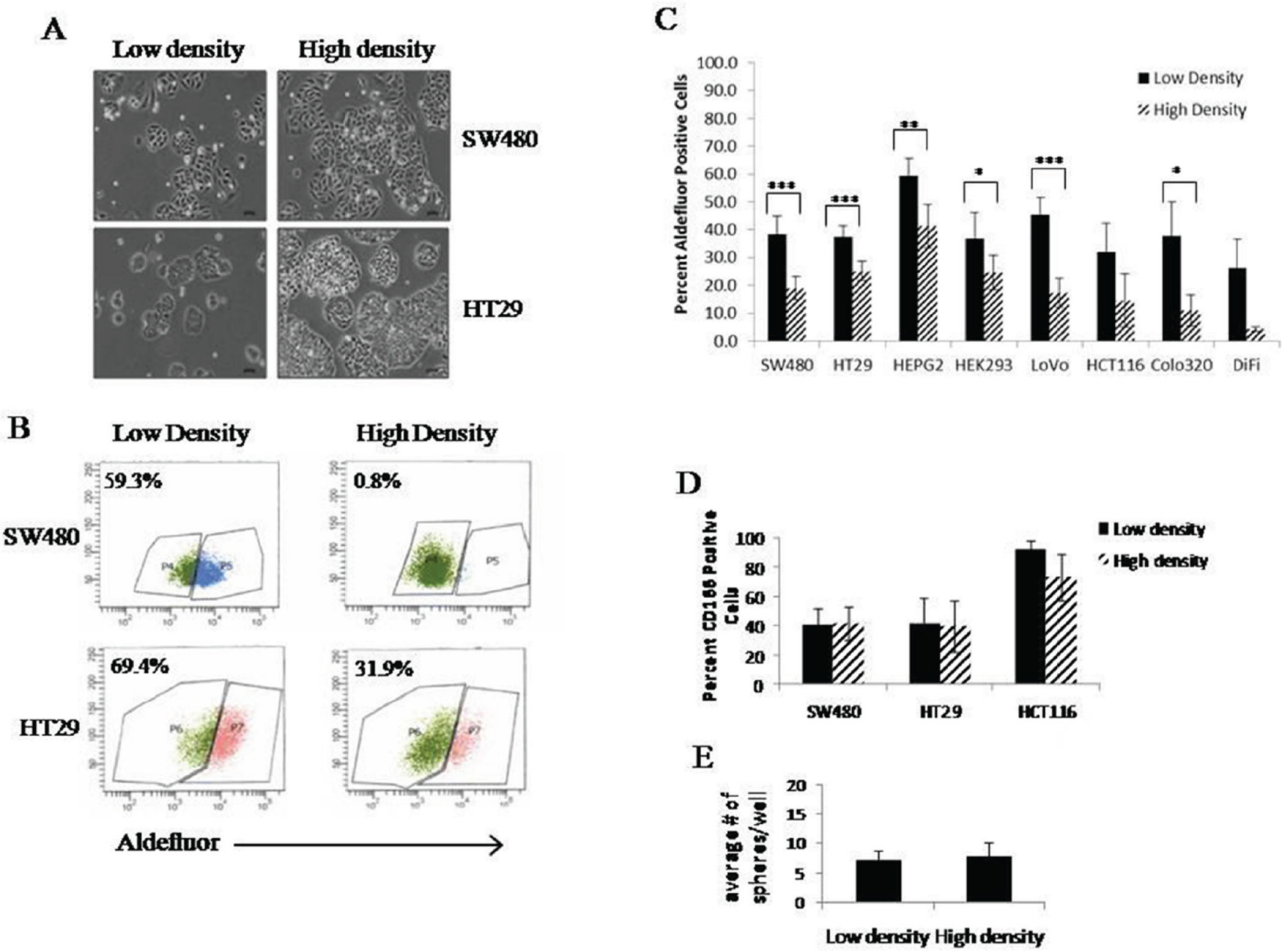
Cell density affects ALDEFLUOR activity without changing other stem cell characteristics. Representative images of HT29 and SW480 cells grown ay low (30–40%) and high (70–80%) cell densities (A). It is important to note that the cells never reach full confluence before analysis. Cells from low and high density were cultured and ALDEFLUOR assay was performed. Representative histograms from one set of analysis on HT29 and SW480 cells grown at low and high density (B). Different colon cancer cell lines were cultured at low and high densities and ALDEFLUOR was performed. The average values of percent positive cells is graphed to show significant changes in ALDEFLUOR positive cells at low and high densities (C). Expression of CD166 on three different colon cancer cell lines grown at low and high densities (D). Cells were grown at low and high density and then plated for colonosphere assay. Graph represents average number of spheres formed under each culture condition (E). All experiments were N=3 except ALDEFLUOR assay was performed with multiple relicates (N=10). *=*p*<0.05, **= *p*<0.01, ***=*p*<0.001.

Our laboratory observed significant variations in the percent of ALDEFLUOR positive cells from one experiment to the next and between scientist to scientist. In order to understand why such large variations occurred, we looked at several different cell lines and plated them at different cell densities (Figure 1A). We found that when most cells were harvested at a lower density (30–40%) they had a higher percent of ALDEFLUOR positive cells. However, when the cells were harvested at a higher density (70–80%), but not confluent, the percent ALDEFLUOR activity was much lower (Figure 1B). This difference between the percent ALDEFLUOR positive cells in low and high density was statistically significant in 6 of the 8 cell lines (Figure 1C). The DiFi and HCT116 did show the same trend with decreased ALDEFLUOR activity in high density cultures, but they did not reach a statistically significant difference in the percent ALDEFLUOR positive cells between the two cell densities (Figure 1C).

While the ALDEFLUOR assay is a commonly used method to identify and isolate stem cells, another marker often used to identify colonic stem cells is CD166. To investigate if the changes we see in ALDEFLUOR activity at different densities correlates with changes in the stem cell population based on another marker, we evaluated the percent of CD166 positive cells in three cell lines at low and high densities. In the three cell lines we studied, none of them showed differences in CD166 expression with cell density (Figure 1D). To further assess changes in the stem cell characteristics based on density differences, we performed a colonosphere assay for self-renewal ability. Cells were cultured under the conditions that yield high or low density, and at that point, the same number of cells derived from each culture condition was dissociated and plated for the sphere formation assay. After 10 days, there was no change in the number or sizes of the spheres formed from either condition (Figure 1E). This indicates that whatever changes are occurring are culture dependent and when cells are removed from these culturing conditions, they do not retain the changes in the ALDH-positive stem cell population.

**Figure 2:**
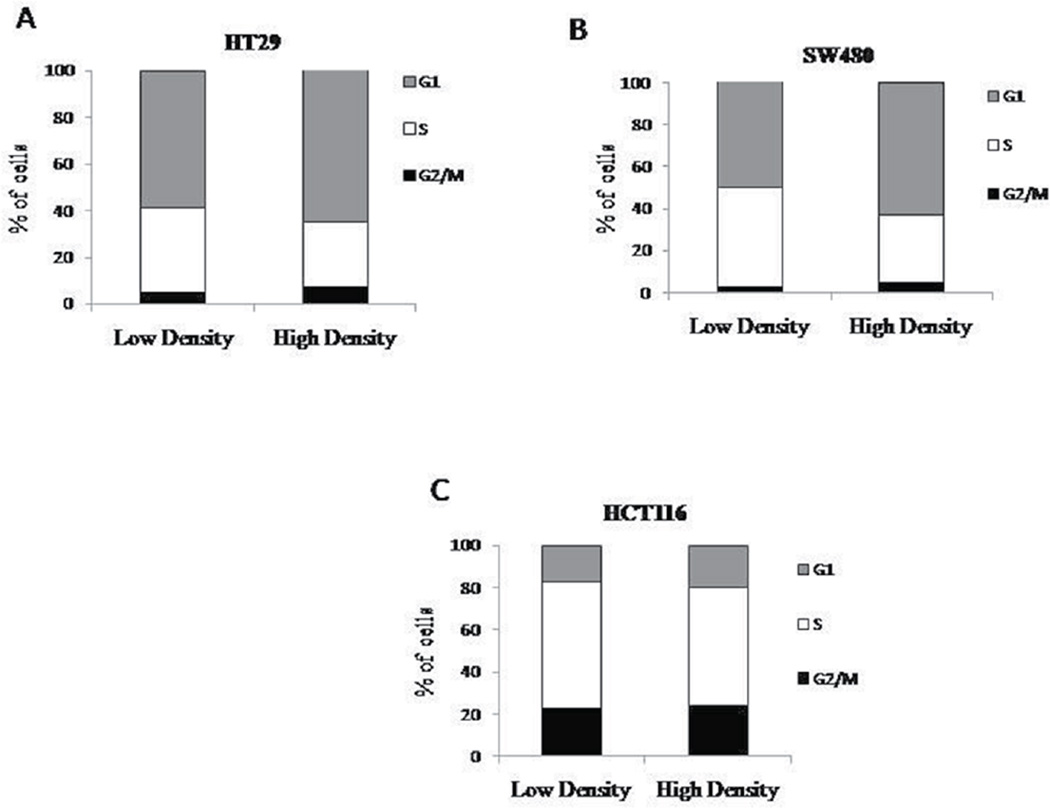
Cell cycle does not change with density. Cells were plated at low and high densities and then fixed before propidium iodide analysis. HT29 (A), SW480 (B), and HCT116 (C) cells were analyzed for different phases of the cell cycle and there was no significant changes seen between each phase (N=3).

The progression of cells through the cell cycle could possibility explain the decreased number of ALDEFLUOR positive cells at high density. Stem cells are believed to have slow cycling times as compared to other cell types found in the colonic epithelium. If the higher ALDEFLUOR positive population observed at low density is a result of increased number of stem cells, then fewer cells present in S phase would correlate with this finding. However, at high density, there is no substantial change in the percent of cells at any of the stages of the cell cycle when compared to the lower density cultures (Figure 2).

**Figure 3:**
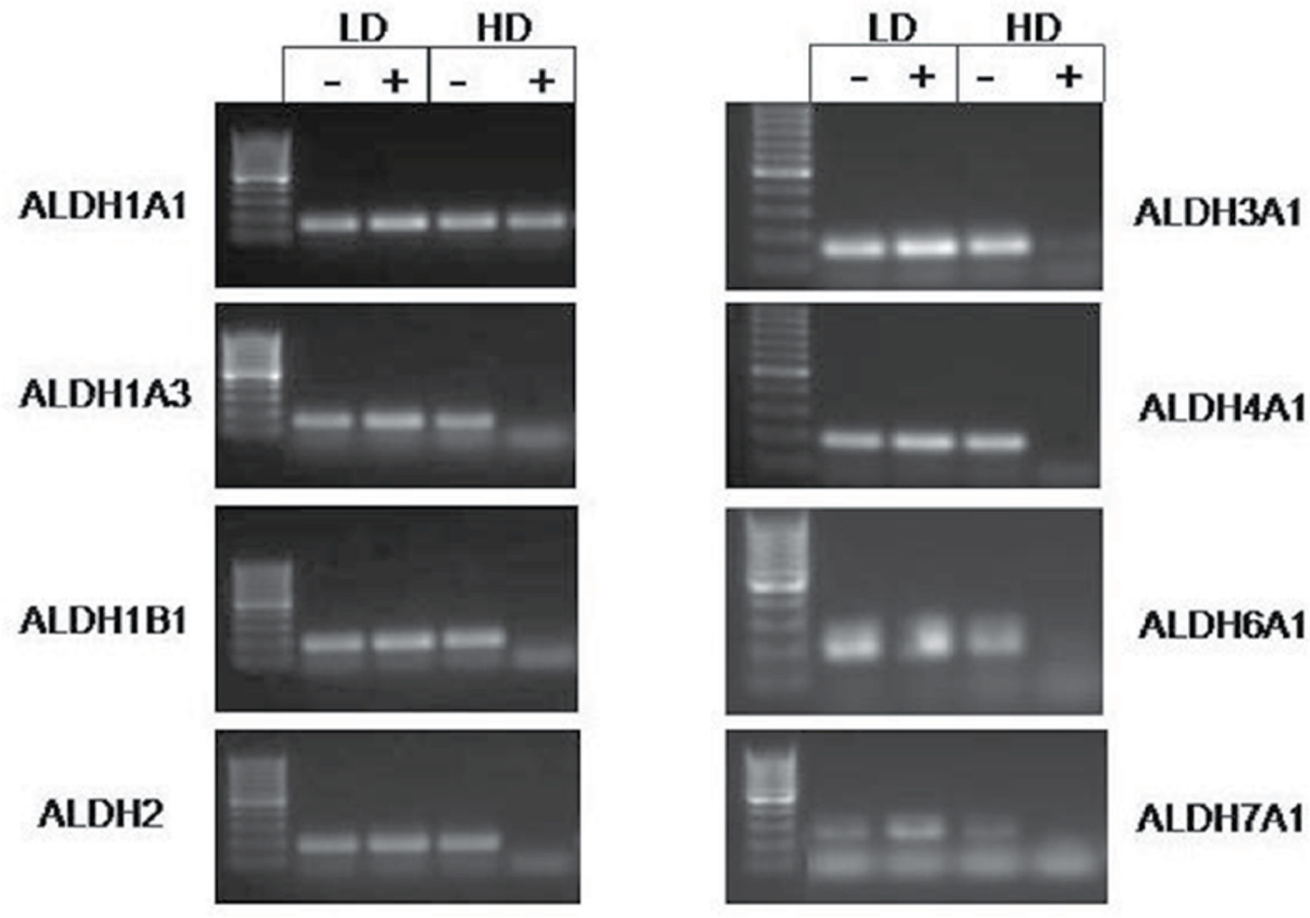
Expression of several ALDH isoforms change with density in HT29 cells. Images are representative of RT-PCR data obtained from multiple experiments. All isoforms shown, except ALDH1A1, demonstrate reduced or absent expression in the high density ALDH positive samples.

Based on these findings described above, we haven’t seen any change to explain why there is a higher percentage of ALDEFLUOR positive cells at lower cell densities. We then surmised that the level of ALDH expression might decrease at high density. In the literature, there are 19 different ALDH isoforms that have been identified. Accordingly, we sorted out the ALDEFLUOR positive and negative populations from cells grown in both the high and low density and performed PCR for each of the 19 isoforms. In HT29 cells, several isoforms were decreased in the high density ALDEFLUOR positive populations (Figure 3). Compared to the high density ALDH negative population, approximately 50% of the isoforms expressed show a decreased presence in the ALDH positive samples. Some of the same isoforms follow this pattern in the SW480 cells as well, but not to the extent as seen in HT29 cells (Figure 3). Based on several different sets of cells that were screened, the expression of the 19 ALDH isoforms is summarized in Table 1. Our results indicate expression of several ALDH isoforms in colorectal cancer becomes decreased at high density.

## DISCUSSION

Large differences were observed in ALDEFLUOR activity between cell cultures grown in low and high densities. We evaluated several different colon cancer cell lines, as well as HepG2 and HEK293 cells, and all lines except DiFi and HCT116 cells, showed a significant difference in ALDEFLUOR activity when cultured at different densities. In the literature, there are many reports of different percentages of ALDH or ALDEFLUOR positive cells in various colon cancer cell lines. In the current study we found SW480 cells have an average of 38% ALDH positive cells in low density growth conditions and 19% ALDH positive cells in high density conditions. In comparison, others have cited the SW480 cell population to have percentages of ALDH positive cells approximately 17.5±0.07% which resemble our values for cultures grown in high density conditions, or 48.3% ALDH positive cells which resemble more of our cultures grown in low density conditions.^3^ HCT116 cells also had a wide range of ALDH positive cells, but while trending to a decreased ALDEFLUOR positivity in high density cultures, HCT116 populations did not reach statistical significance in our analysis. However, in the literature, there is a large range of HCT116 ALDH positive cells (~4.0–49% ALDH positive) with no clear mention of culture density conditions.^1–4^ Overall, these reports illustrate the discrepancy between the ALDEFLUOR assay and quantification of stem cell populations. Our data herein shows a significant difference between the population of stem cells based on low and high density growth cultures. It is of interest to note that the experiments conducted in this study were done within ten passages and based on our results there was no correlation between passage number and ALDEFLUOR activity. Our findings suggest a standard needs to be set, according to cell density, when performing experiments in order to be able to compare experimental results on stem cell populations based on the ALDEFLUOR assay.

After discovering this change in ALDH positive cells in low and high density cultures, we wanted to see if this difference translated to other colon cancer stem cell markers. Research suggests that there are several subpopulations of stem cells that reside within a tissue.^25^ Therefore, we looked specifically at CD166 expression since it is known that this is another marker for identification of colon cancer stem cells.^26,27^ We see that CD166 and ALDH1 identify different sub-populations of colon CSCs as immunostaining marks distinct subsets of cells. Interestingly, the density-based changes we noted to be associated with ALDEFLUOR assay are unique to the sub-population of ALDH-positive cells, but not to the CD166-positive cells. Thus, the stem cells marked by high ALDEFLUOR activity appear to be sensitive to changes in cell density, but the sub-population of stem cells marked by CD166 expression is not affected.

Since we saw a significant difference in ALDEFLUOR activity in low and high density cultures, we examined sphere formation to corroborate the effect of density on the stem cell property of self-renewal of these cells. Colonosphere assays showed that no significant change occurred in sphere formation or sphere size with cell density.

We then measured the distribution of cells in the different phases of the cell cycle between cells grown in low and high cell density. There was no statistically significant change in the percent of cells in each phase of the cell cycle with different density cultures. It is important to note that the cell cycle analysis shows cells are still proliferating at high density. It could be possible that the cells are distributed similarly throughout the cell cycle, but that the time it takes the cells to transverse the cycle is slower.

Up to this point we had not seen any changes between cell cultures grown in low and high density other than the change in ALDEFLUOR assay. To try to discern what might cause these observed differences, we grew cells to low and high density and then sorted the ALDEFLUOR positive and ALDEFLUOR negative populations to assess changes in the ALDH messenger RNA (mRNA) level. Although not widely published, researchers have observed changes in gene expression when cells are cultured at different densities.^8^ One group has looked at changes in gene expression in mesenchymal stem cells. They observed that less dense cultures upregulate genes involved in proliferation and when the cultures become more confluent these genes are down-regulated and genes involved in secretion of cytokines are upregulated.^7^ It is possible that this is similar in our cultures. At lower densities, genes involved in proliferation could be upregulated and increased stem cell population size could occur to maintain the proliferative capacity of the culture. At higher cells densities, while still not yet confluent, cells could begin the process of maturation, thus causing the stem cell population size to contract.

In order to look at changes at the mRNA level of ALDEFLUOR positive and negative cells grown in low and high cell densities, we profiled the entire spectrum of ALDH isoforms. There are 19 different isoforms of ALDH and not one specific type of isoform is linked directly to ALDEFLUOR assay, although the manufacturer Stem Cell Technologies Inc. reports that the assay was optimized for the ALDH1A1 isoform. However, based on our PCR data, there may be other isoforms responsible for the ALDEFLUOR activity in colon cancer stem cells. Indeed, our study demonstrated the involvement of several isoforms previously not identified in colon cancer. While data exists linking ALDH1B1 to colon cancer as a potential biomarker^20^ and modulator of the Wnt/β-catenin, Notch and PI3K/Akt signaling pathways,^28^ few other studies have looked specifically at this isoform. Changes in the ALDH1A3 isoform is interesting, as this isoform has been linked to many other cancer types and often follows the same pattern of ALDH1A1. In fact, ALDH1A3 was identified as the dominant isoform in breast cancer responsible for ALDEFLUOR activity.^17^ In colon cancer cell lines, ALDH1A3 is upregulated in chemoresistant lines^29^ and its regulation in chemoresistant lines may be due to changes in expression.^30^ While there is no data, to our knowledge, showing any links between ALDH4A1, ALDH6A1, and ALDH7A1 expression and colon cancer, our findings show changes in expression of these three isoforms at the mRNA level. Thus, it appears that the activity detected by the ALDEFLUOR assay may be attributed to several different isoforms. It is possible that identification of these other isoforms in CSCs will allow us other means to distinguish colon CSCs from normal stem cells.

## CONCLUSION

Our study discovered a considerable effect of cell density on the degree of ALDH activity using the ALDEFLUOR assay. When cells are cultured in either low or high density there is a significant decrease in ALDH activity, but no drastic changes in CD166 positive cells, sphere formation, or cell cycle distribution. The fact that most of the cancer cell lines in this study showed a significant decrease in ALDH activity when cultured at high density, we surmised there must be a difference in the specific isoform of ALDH that is expressed or not expressed. Indeed, our study identified several unique ALDH isoforms that are differentially expressed in ALDH positive cells that were grown at different densities. Moreover, our findings indicate that there are different ALDH isoforms expressed that have not been previously linked to colorectal cancer. Results from our study could open up new approaches toward targeting colon CSCs. Effective stem-cell-targeted treatments may result, not from the targeting of a single isoform, but multiple ALDH isoforms. Targeting and destroying different sub-populations of CSCs may necessitate inhibiting multiple ALDH isoforms, resulting in growth suppression and lowering the tumor’s self-renewal ability.

## Figures and Tables

**Table 1:** Expression of ALDH isoforms in HT29 and SW480 cells. Reverse Transcriptase PCR was performed on RNA isolated from ALDH positive and negative populations sorted from high and low density cultures. Particularly in the HT29 cell line, some isoforms are absent in the high density ALDH positive fraction.

HT29 cells																			
	1A1	1A2	1A3	1B1	1L1	1L2	2	3A1	3A2	3B1	3B2	4A1	5A1	6A1	7A1	8A1	9A1	16A1	18A1
LD−	+++	+	+++	+++			+++	+++	+++	+++	+	+++		+++	+++		+++	++	+++
LD+	+++		+++	+++			+++	+++	++	+++	++	++		+++	+++		+++	++	+++
HD−	+++	++	+++	+++			+++	+++	+++	+++	++	+++		++	+++		+++	+++	+++
HD+	+++		+	+			+	+	+++	+++		+		+	++		+++		+++
SW480 cells																			
	1A1	1A2	1A3	1B1	1L1	1L2	2	3A1	3A2	3B1	3B2	4A1	5A1	6A1	7A1	8A1	9A1	16A1	18A1
LD−			+++	++++		+	+++	++++	++++	++++		+++	+	+++	+++		++++	++	+++
LD+	+		++++	++++		+	++++	++++	++++	++++		++++	++	++++	++++		++++	++	++++
HD−	++	+++	+++	+++		++	++++	+++	+++	+++		+++	+++	+++	+++		+++	++	+++
HD+	+	+	+++	++++		+	++++	++++	++++	++++		+++	+++	++++	++++		++++	++++	++++

Plus (+) signs indicate presence of a band for each replicate. Three replicates were performed for the HT29 cell line and four replicates were performed for the SW480 cell line.

## ABBREVIATIONS

CSCs: Cancer stem cells
ALDH: Aldehyde dehydrogenase
BAAA: BODIPY-Aminoacetaldehyde
DEAB: Diethylaminobenzaldehyde
ATRA: All-Trans-Retinoic Acid
ATCC: American Type Culture Collection
FBS: Fetal Bovine Serum
HepG2: Hepatocellular carcinoma
HEK293: Human embryonic kidney cells
DMEM: Dulbecco’s Modified Eagle’s Medium
EDTA: (Ethylenedinitrilo)tetraacetic acid
EGF: Epidermal Growth Factor
CTCR: Center for Translational Cancer Research
RNA: Ribonucleic acid
DNA: Deoxyribonucleic acid
DNAse: Deoxyribonuclease
TECAN: Tecan Group Ltd
PCR: Polymerase Chain Reaction
